# Association between DRD2 and ANKK1 polymorphisms with the deficit syndrome in schizophrenia

**DOI:** 10.1186/s12991-020-00289-0

**Published:** 2020-06-17

**Authors:** Anna Michalczyk, Justyna Pełka-Wysiecka, Jolanta Kucharska-Mazur, Michał Wroński, Błażej Misiak, Jerzy Samochowiec

**Affiliations:** 1 Department of Psychiatry, Pomeranian Medical University, 26 Broniewskiego Street, 71-460 Szczecin, Poland; 2 Department of Genetics, Wroclaw Medical University, 1 Marcinkowskiego Street, 50-368 Wroclaw, Poland

**Keywords:** Schizophrenia, Deficit syndrome, Family history of schizophrenia, Gene polymorphisms, DRD2, ANKK1

## Abstract

**Background:**

The clinical course of schizophrenia varies among patients and is difficult to predict. Some patient populations present persistent negative symptoms, referred to as the deficit syndrome. Compared to relatives of non-deficit schizophrenia patients, family members of this patient population are at an increased risk of developing schizophrenia. Therefore, the aim of this study was to search for genetic underpinnings of the deficit syndrome in schizophrenia.

**Methods:**

Three SNPs, i.e., rs1799732 and rs6276 located within DRD2, and rs1800497 within ANKK1, were identified in the DNA samples of 198 schizophrenia probands, including 103 patients with deficit (DS) and 95 patients with non-deficit schizophrenia (NDS). Results: No significant differences concerning any of the analyzed polymorphisms were found between DS and NDS patients. However, significant links were observed between family history of schizophrenia and the deficit syndrome, G/G genotype and rs6276 G allele. In a separate analysis, we identified significant differences in frequencies of rs6276 G allele between DS and NDS patients with family history of schizophrenia. No significant associations were found between DRD2 and ANKK1 SNPs and the age of onset or schizophrenia symptom severity.

**Conclusions:**

The results of our preliminary study fail to provide evidence of associations between DRD2 and ANKK1 polymorphisms with the deficit syndrome or schizophrenia symptom severity, but suggest potential links between rs6276 in DRD2 and the deficit syndrome in patients with hereditary susceptibility to schizophrenia. However, further studies are necessary to confirm this observation.

## Background

Schizophrenia is a common mental disorder with a multifactorial background, and a lifetime prevalence of about 0.5–1% [1]. Its course is varied and difficult to predict. Some patients experience a single psychotic episode, while others report alternating periods of exacerbation and remission, or gradual functional deterioration from the very onset, with permanent negative symptoms. Early and precise recognition and prognosis, especially regarding disease progression enables timely implementation of appropriate preventive measures [2, 3]. Based on observations of Carpenter et al. [4], it was proposed that enduring negative symptoms, which are present during or in-between episodes of exacerbation of positive symptoms, and independently of social isolation, antipsychotic treatment, or depressive symptoms, should be distinguished as another schizophrenia subtype and called the “deficit syndrome”. Although none of the available classifications of diseases include the term “deficit schizophrenia”, the ICD-11 and DSM-5 identify schizophrenia with dominant negative symptoms [5, 6]. The deficit syndrome is linked with a stable progression of negative symptoms, observed from illness onset throughout the course of the disease [7–10], and its incidence in patients with chronic schizophrenia is estimated at 25–30% [11]. Evidence from family, twin and adoption studies highlights the key role of genetic factors in the development of schizophrenia [12] and its heritability of up to 80% [13]. Associations with family history were also found for the deficit syndrome [11].

Despite years of research, pathogenesis of schizophrenia is yet to be fully understood, but dysregulation of dopaminergic neurotransmission seems to be somehow implicated in its development. Positive symptoms are hypothesized to result from excessive dopamine activity in the mesolimbic pathway, while the negative symptoms and cognitive impairment to stem from reduced dopamine activity in the mesocortical pathway [14]. Findings suggest elevated density of dopamine D2 receptor (DRD2) in the brains of schizophrenic patients and most antipsychotic agents act as DRD2 antagonists [15–17]. However, studies on the effect of antipsychotic drugs on negative symptoms yield inconsistent results. According to some, antipsychotic medication may trigger or worsen negative symptoms in both clinical [18] and non-clinical samples [19–21], while others suggest no clinically meaningful effect on negative symptoms [22]. According to de Haan et al. [23], negative symptoms may be related to striatal dopamine D(2) receptor occupancy, but other studies fail to replicate such findings [24, 25]. Brito-Melo et al. [26] showed an association between increased expression of DRD2 in peripheral blood T-cells, higher scores in the Brief Psychiatric Rating Scale (BPRS) and the Positive and Negative Syndrome Scale (PANSS). Liu et al. [27] also found a relationship between DRD2 expression and negative symptoms, observing a positive correlation between the deficit syndrome severity and DRD2 expression in peripheral blood leukocytes of chronic schizophrenia patients receiving clozapine treatment.

Although a linkage was reported between the DRD2 genomic location (11q22–11q23) and schizophrenia [28], the results of association studies of particular polymorphisms remain inconsistent, due possibly to relatively small sample sizes, multifactorial underpinnings of schizophrenia and heterogeneous study groups [29]. In this study, we decided to investigate whether common functional single nucleotide polymorphisms (SNPs) located within DRD2 (rs1799732, rs6276) and ankyrin repeat and kinase domain containing 1 (ANKK1; rs1800497) are associated with the deficit syndrome or symptom severity in schizophrenic patients.

Rs1799732 (-141C Ins/Del) is a functional SNP located in the 5′-promoter region of DRD2, affecting striatal dopamine receptor density [30] and likely associated with schizophrenia [31–37], although not all studies confirm this association [38–40]. Himei et al. report a link between rs1799732 and PANSS-positive symptom score in schizophrenic patients [41]. In turn, according to Ohara et al. [31], it is associated with the age of onset, but not the PANSS scores in this patient population. Lencer et al. [42] demonstrate a link between rs1799732 and motor and cognitive function in first-episode psychosis. On top of that, it is also associated with antipsychotic efficacy [43, 44], antipsychotic-induced weight gain [45], neuroleptic-induced extrapyramidal adverse effects [46], personality traits [47, 48], sexual dysfunction in male schizophrenia [49], suicide attempts [50, 51] and alcoholism [52].

Rs6276 is a putative functional polymorphism located in 3′UTR region of DRD2 gene. To date, there seems to be a paucity of research examining its links with schizophrenia, but it has been associated with total and spatial insight problem solving [53], creative potential determined by verbal and figural divergent thinking tests [54], susceptibility to delirium [55], alcohol consumption [56], acute pain severity [57] and human longevity [58].

Rs1800497, previously known as DRD2 Taq 1A, is a functional polymorphism in adjacent ankyrin repeat and kinase domain containing 1 (ANKK1) gene, affecting dopamine D2 receptor binding [59]. Findings concerning its links with schizophrenia are somewhat inconsistent [29, 36, 60–63]. There is evidence of its associations with the age of schizophrenia onset [64], antipsychotic-induced weight gain [65], antipsychotic treatment response [66] and mood disorders [67, 68]. Takeuchi et al. [69] demonstrated its effect on emotional intelligence, which is, albeit indirectly, believed to also affect divergent thinking and motivation. Interestingly, haplotypes of rs1079597-rs1800497 were found to be associated with improvements in negative symptoms after amisulpride treatment, but there was no significant finding concerning the polymorphism itself [70].

Despite many studies linking dopamine receptors and their genetic polymorphisms with schizophrenia and its clinical manifestation, including the age of onset, cognitive functions, positive and negative symptom severity, treatment response and adverse effects, there is still a relative paucity of research investigating direct associations between DRD2 polymorphisms and the deficit syndrome. Therefore, the aim of this study is to fill this gap and examine the effect of DRD2 and ANKK1 polymorphisms on the deficit syndrome.

## Methods

### The sample

**Fig. 1:**
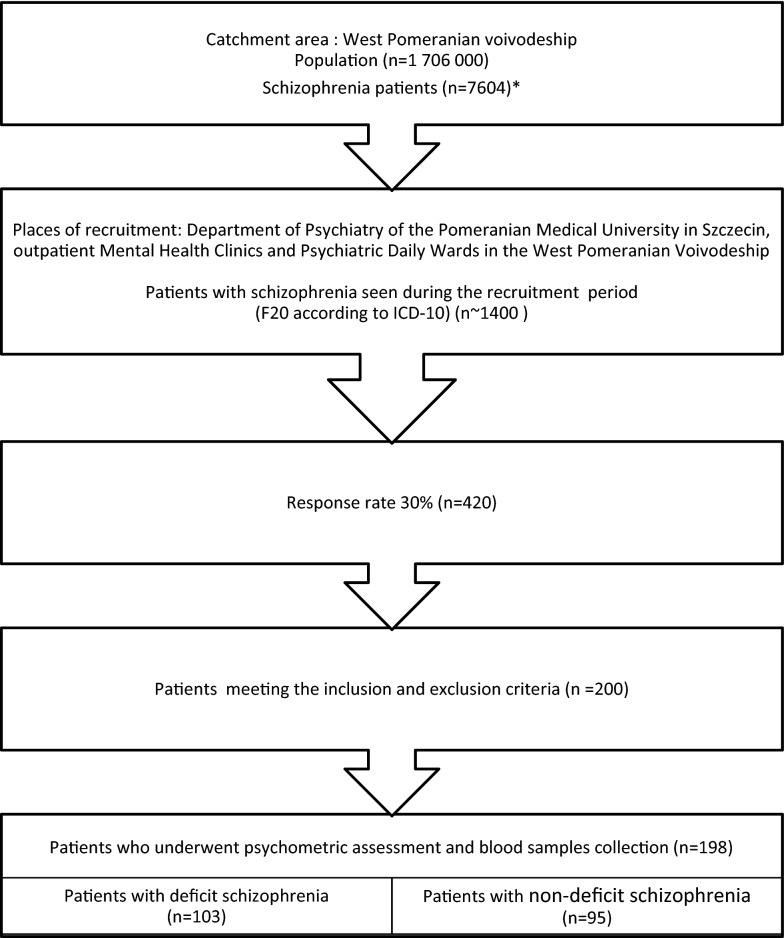
Strobe flowchart of the recruitment process

Our sample consisted of 198 Caucasian patients with schizophrenia, recruited in the Department of Psychiatry of the Pomeranian Medical University in Szczecin, Outpatient Mental Health Clinics and Psychiatric Day Wards in West Pomeranian voivodeship in the years 2011–2015 (Fig. 1).

Inclusion criteria were: age of 18–60 years, diagnosis of schizophrenia (F20 according to the ICD-10 [71–73]), disease duration of > 18 months, comprehension of test procedures and an informed consent to participate in the study. Exclusion criteria comprised: psychiatric comorbidity (including affective disorders), dementia or significant brain damage, epilepsy, addiction to alcohol or other psychoactive substances (excluding nicotine) and somatic conditions (cancer, chronic cardiovascular, respiratory, digestive, excretory or hormonal disorders). All recruitment procedures were carried out by two specialist psychiatrists. All participating clinicians were trained in the use of psychometric scales and had valid Good Clinical Practice certifications. The necessary calibrations were performed before baseline—the research team practiced implementation of study procedures on a standard patient, commenting on the results and setting endpoint criteria. Diagnostic assessment was performed using standardized research tools—the Mini-international neuropsychiatric interview [74], Operational Criteria Checklist for Psychotic Illness and Affective Illness (OPCRIT) [75], and a self-designed questionnaire. Psychometric assessment included validated Polish versions of the Positive and Negative Syndrome Scale (PANSS) [76–79], the Clinical Global Impression-Schizophrenia scale (CGI-SCH) [80, 81] and the Mini-Mental State Examination (MMSE) [82, 83]. In the PANSS, the positive (P1–P7), negative (N1–N7) and general (G1–G16) symptoms were rated on a 0–6 scale, based on the severity of each symptom. The CGI-SCH was used to assess the severity of schizophrenia across overall, positive, negative, depressive, and cognitive subdomains. Each symptom was scored on a scale from 1 to 7 [(1) norm, lack of dysfunction, (2) minimally ill, (3) slightly ill, (4) moderately ill, (5)-clearly ill, (6) seriously ill, 7-extremely ill). The MMSE was used to screen for cognitive impairment. A score of 27–30 suggested no cognitive impairment, 24–26—cognitive impairment without dementia, 19–3—mild dementia, 11–18—moderate dementia, and ≤ 10 severe dementia. None of the patients scored < 24 points. All patients received typical or atypical antipsychotics and were treated according to the psychopharmacological guidelines for the treatment of schizophrenia [84–87].

Based on clinical symptoms, medical history of functional impairment, and the Schedule for the Deficit Syndrome (SDS) outcome, the sample was divided into two subgroups: (1) patients with deficit (DS), and (2) non-deficit schizophrenia (NDS) [88–92]. The SDS ratings were made on a five-point scale, based on the primary or secondary character, persistence and intensity of each of the following six negative symptoms: restricted affect, diminished emotional range, poverty of speech, curbing of interests, diminished sense of purpose, diminished social drive. Patients with SDS score of ≥ 2 and two or more primary and permanent (i.e., lasting at least 12 months) negative symptoms were qualified to the DS subgroup, while all others comprised the NDS subgroup. The two groups differed in gender proportions, family history of schizophrenia (in first or second degree relatives), duration of longest relapse, and their MMSE, PANSS, CGI-SCH and SDS scores (Table 1).

**Table 1:** Demographic and clinical characteristics of the study subgroups

Parameter	DS	NDS	*X*^2^	*Z*	*p*
Women *n (%)*	43 (42)	58 (61)	7.37		*0.007*
Age (years) *mean ± SD (median)*	41 ± 11 (40)	39 ± 11 (36)		− 1.46	0.143
Higher education *n (%)*	18 (17)	18 (19)	0.07		0.788
Cohabitation *n (%)*	32 (31)	29 (31)	0.01		0.934
Psychiatric illness in the family *n (%)*	41 (40)	33 (35)	0.46		0.496
Neurological illness in the family *n (%)*	17 (17)	13 (14)	0.27		0.603
Schizophrenia in the family *n (%)*	30 (29)	15 (16)	4.83		*0.028*
Duration of schizophrenia (years) *mean ± SD (median)*	14 ± 9 (13)	13 ± 10 (10)		− 1.73	0.084
Age of onset (years) *mean ± SD (median)*	27 ± 8 (24)	26 ± 8 (24)		− 0.30	0.761
Time delay from onset to diagnosis *n (%)*					
< 1 month	49 (48)	48 (51)			
1–6 months	23 (22)	21 (22)	0.22		0.897
> 6 months	31 (30)	26 (27)			
Withdrawal of symptoms after neuroleptics *n (%)*	97 (94)	93 (98)	1.76		0.184
Number of psychiatric hospitalizations *mean ± SD (median)*	6.7 ± 5.8 (5)	6.4 ± 6.8 (4)		− 1.14	0.253
Longest episode (weeks) *mean ± SD (median)*	33 ± 62 (14)	19 ± 27 (12)		− 2.31	*0.021*
Clinical course *n (%)*			0.31		0.855
Episodes of relapses and remissions	78 (76)	82 (86)			
Chronic course	10 (10)	8 (8)			
Chronic course with social withdrawal	14 (14)	5 (5)			
MMSE mean ± SD *(median)*	28 ± 2 (28)	28 ± 2 (29)		2.29	*0.022*
PANSS *mean ± SD (median)*					
Total	42 ± 18 (41)	22 ± 19 (17)		− 7.08	*< 0.001*
Positive	6 ± 5 (6)	5 ± 5 (3)		− 2.72	*0.006*
Negative	16 ± 6 (15)	7 ± 6 (5)		− 9.01	*< 0.001*
General	19 ± 10 (18)	10 ± 9 (8)		− 6.37	*< 0.001*
CGI-SCH mean ± SD (median)					
Total	14 ± 5 (14)	9 ± 4 (8)		− 7.79	*< 0.001*
Positive	2.3 ± 1.3 (2)	1.8 ± 1.0 (1)		− 2.23	*0.026*
Negative	3.8 ± 1.3 (4)	2.1 ± 1.2 (2)		− 8.10	*< 0.001*
Depressive	2.3 ± 1.1 (2)	1.4 ± 0.6 (1)		− 6.73	*< 0.001*
Cognitive	2.8 ± 1.1 (3)	1.8 ± 0.9 (2)		− 6.10	*< 0.001*
Total severity of schizophrenia	3.2 ± 1.1 (3)	2.0 ± 1.0 (2)		− 7.35	*< 0.001*
SDS *mean ± SD (median)*	15 ± 5 (15)	6 ± 6 (6)		− 8.99	*< 0.001*

### Genetic tests

DNA was extracted from leukocytes using a salting out procedure [93]. In thus obtained genetic material, we identified three SNPs: rs1799732 and rs6276 located within DRD2 and rs1800497 within ANKK1. The tests were carried out using the real-time polymerase chain reaction (real-time PCR) technique, with the use of LightSNiP probes (TIB MOLBIOL GmbH) and melting curve analysis on the LightCycler 2.0 instrument (Roche Diagnostics). All assays were carried out in the Psychiatrics Genetic Laboratory at the Department of Psychiatry of the Pomeranian Medical University in Szczecin. Genotyping was performed blind to study group assignment (DS/NDS).

### Statistical analysis

Statistical analysis was performed using Statistica 13 software (StatSoft, Inc.). Sample size was calculated using Statistica software for sample size calculation (two-sample Z test). We assumed a power of 0.90 and *α* = 0.05 to detect a medium effect size for differences in allelic proportions between DS and NDS patients for all analyzed SNPs. As per proportion estimation, we considered minor allele frequency of particular SNPs in the European population (https://www.ncbi.nlm.nih.gov/projects/SNP/), and the expected difference between arcsine transformed proportions (h) of 0.5 in the investigated groups, which is considered a medium effect size [94]. According to our calculations, the minimum number of patients was 87 for each of the subgroups, 174 in total. Therefore, the collected material was sufficient to perform all planned analyses. The Hardy–Weinberg equilibrium was assessed using a calculator (http://www.dr-petrek.eu/documents/HWE.xls, access on 16.11.2018). Pearson’s *Χ*^2^ test was used to analyze differences in distribution of genotypes and alleles between the two groups. For small sample sizes (if frequency of one of the genotypes was < 10), significant differences were verified using the Yates correction. Most of the analyzed variables deviated from the normal distribution in the Shapiro–Wilk test, therefore the Mann–Whitney U test and Kruskal–Wallis test were performed to analyze associations between genotypes, patient subgroups and the quantitative variables. In all analyses, statistical significance was set at *p* > 0.05.

## Results

For all analyzed SNPs, the observed genotype distributions in the study group were consistent with the Hardy–Weinberg equilibrium (*χ*^2^ < 0.16, *p* > 0.68).

Direct comparisons did not show any significant differences in genotype or allele distributions between patients with deficit and non-deficit schizophrenia for any of the studied polymorphisms (Table 2).

**Table 2:** Comparison of genotype and allele distributions of DRD2/ANKK1 SNPs between schizophrenic patients with (DS) and without (NDS) the deficit syndrome

Group	*n*	Genotypes *N* (%)			*χ*^2^	*p*	Alleles *N* (%)		*χ*^2^	*p*
rs1799732										
		C/C	C/del	del/del	3.86	0.145	C	del	1.73	0.188
DS	103	77 (75)	25 (24)	1 (1)			179 (87)	27 (13)		
NDS	95	80 (84)	13 (14)	2 (2)			173 (91)	17 (9)		
rs6276										
		A/A	A/G	G/G	2.98	0.226	A	G	1.18	0.277
DS	103	42 (41)	44 (43)	17 (17)			128 (62)	78 (38)		
NDS	95	41 (43)	46 (48)	8 (8)			128 (67)	62 (33)		
rs1800497										
		C/C	C/T	T/T	1.57	0.457	C	T	0.002	0.966
DS	102	68 (67)	29 (28)	5 (5)			165 (81)	39 (19)		
NDS	95	61 (6)]	32 (34)	2 (2)			154 (81)	36 (19)		

Due to significant differences in gender proportions between the DS and NDS groups, we decided to perform an additional separate subgroup analysis. However, we found no significant gender-related differences in genotype or allele frequencies between DS and NDS patients (*p* > 0.1). No differences in genotype or allele frequencies were found between women and men in the entire sample or the DS and NDS subgroups (*p* > 0.1).

In our sample, the G/G genotype and rs6276 G allele were significantly more frequent in patients with family history of schizophrenia than in those without schizophrenia in the family (Table 3). Likewise, a separate subgroup analysis of DS patients showed that the G/G genotype and rs6276 G allele were significantly more frequent in patients with schizophrenia in the family, compared to those with negative family history (30% vs 10%, *χ*^2^ = 5.44, *p* = 0.020 and 55% vs 31%, *χ*^2^ = 10.12, *p* = 0.0015, respectively). These associations proved significant even after the Yates correction for small sample sizes (*χ*^2^ = 4.14, *p* = 0.041 and *χ*^2^ = 9.14, *p* = 0.0025, respectively). In the NDS group, there were no significant associations between family history of schizophrenia and genotype or allele frequencies for any of the analyzed SNPs.

**Table 3:** Comparison of genotype and allele distribution of DRD2/ANKK1 SNPs between schizophrenic patients with positive and negative family history of schizophrenia

Family history of schizophrenia	*n*	Genotypes *N* (%)			*χ*^2^	*p*	Alleles N (%)		*χ*^2^	*p*
rs1799732										
		C/C	C/del	del/del	5.71	0.058	C	del	2.13	0.144
Positive	45	31 (69)	14 (31)	0 (0)			76 (84)	14 (16)		
Negative	150	123 (82)	24 (16)	3 (2)			270 (90)	30 (10)		
rs6276										
		A/A	A/G	G/G	7.22	*0.027*	A	G	6.84	**0.009**
Positive	45	13 (29)	22 (49)	10 (22)			48 (53)	42 (47)		
Negative	150	69 (46)	67 (45)	14 (9)			205 (68)	95 (32)		
rs1800497										
		C/C	C/T	T/T	0.94	0.625	C	T	0.94	0.333
Positive	45	32 (71)	12 (27)	1 (2)			76 (84)	14 (16)		
Negative	149	95 (64)	48 (32)	6 (4)			238 (80)	60 (20)		

The DS was more frequent in patients with family history of schizophrenia than in those with negative family history (67% vs 48%, *χ*^2^ = 4.83, *p* = 0.028). A separate analysis of patients with family history of schizophrenia showed significant differences in rs6276 G allele distributions between DS and NDS patients (55% vs 30%, *χ*^2^ = 5.02, *p* = 0.0025, after Yates correction *χ*^2^ = 4.07, *p* = 0.044). In addition, rs6276 was also associated with family history of mental illness (Table 4). We did not find any significant associations between DRD2 and ANKK1 SNPs and other analyzed clinical parameters (Table 4).

**Table 4:** Associations between DRD2 and ANKK1 gene polymorphisms and selected clinical parameters in schizophrenic patients (*N* = 198)

Parameter	rs1799732		rs6276		rs1800497	
	*χ*^2^* *H*^#^	p	*χ*^2^* *H*^#^	*p*	*χ*^2^* *H*^#^	*p*
Psychiatric illness in the family	0.08	0.777	9.54	*0.002*	1.98	0.159
Neurological illness in the family	0.06	0.807	1.08	0.299	2.52	0.112
Age of onset (years)	0.85	0.653	1.01	0.603	0.98	0.611
Time delay from onset to diagnosis	4.75	0.093	0.03	0.987	2.41	0.299
< 1 month						
1–6 months						
> 6 months						
Withdrawal of symptoms after neuroleptics	0.99	0.321	1.57	0.211	1.61	0.204
Number of psychiatric hospitalizations	2.97	0.226	0.41	0.814	3.65	0.161
Longest episode (weeks)	1.25	0.535	3.90	0.142	0.52	0.770
Clinical course	0.01	0.996	2.00	0.368	1.04	0.596
Episodes of relapses and remissions						
Chronic course						
Chronic course with social withdrawal						
MMSE	3.07	0.216	2.18	0.337	0.47	0.789
PANSS						
Total	2.51	0.285	0.33	0.846	0.70	0.705
Positive	4.83	0.090	0.67	0.717	0.76	0.684
Negative	1.32	0.517	1.34	0.512	2.64	0.267
General	2.37	0.306	0.02	0.992	0.50	0.779
CGI-SCH						
Total	0.70	0.703	0.38	0.828	0.62	0.734
Positive	0.62	0.734	1.54	0.462	1.80	0.406
Negative	0.40	0.819	0.97	0.615	0.83	0.66
Depressive	0.93	0.627	0.82	0.66	1.73	0.421
Cognitive	0.66	0.718	0.73	0.693	2.42	0.298
Total severity of schizophrenia	0.83	0.66	0.27	0.872	0.03	0.987
SDS	0.16	0.924	0.85	0.654	1.78	0.411

* *χ*^2^ coefficient for allele comparison in Pearson’s test (genotype comparisons were abandoned due to the small numbers expected for certain genotypes)

^#^H coefficient, Kruskal–Wallis test for genotype comparison

## Discussion

In this study, we analyzed the influence of DRD2 and ANKK1 gene polymorphisms on the disease phenotype in schizophrenic patients. Given that most antipsychotic drugs act as DRD2 antagonists, and although the results of meta-analyses remain inconclusive [29, 36, 95–98], about 35% of case–control studies suggest that at least one SNP in DRD2 is significantly associated with schizophrenia [99], we assumed that its biological underpinnings are complex and that the differences in clinical phenotype may, at least in part, result from polymorphisms in DRD2 gene. It was demonstrated that the family members of DS patients have a 1.75 times greater risk of schizophrenia compared to family members of NDS patients, and that the presence of DS in the family increases the risk of the deficit syndrome in schizophrenia patients [11, 100, 101]. Therefore, we decided to check if the presence of the “deficit syndrome” in schizophrenic patients is associated with distinct genetic factors.

Overall, we found no associations between the presence of the deficit syndrome and any of the analyzed SNPs in the entire sample of schizophrenic patients. We also found no associations with any other analyzed clinical parameters, which suggests a lack of associations between DRD2 polymorphisms and the clinical course of schizophrenia.

The only positive result was the observation that DRD2 rs6276 was significantly more frequent in patients with family history of schizophrenia. In addition, a separate analysis of this patient subgroup showed that the DRD2 rs6276 G allele was significantly more frequent in patients with DS compared to NDS, which may suggest its associations with inherited susceptibility to schizophrenia and its DS phenotype. This observation, however, needs to be further confirmed in family studies.

To the best of our knowledge, no previous studies investigated associations between SNP rs6276 in DRD2 and the phenotype of schizophrenia, but Liu et al. showed that DRD2 mRNA expression levels in chronic schizophrenia patients on clozapine treatment were correlated with severity of the deficit syndrome [27]. Chien et al. demonstrated significant associations between some SNPs and haplotypes of DRD2 (not analyzed in our study), negative symptom severity and sustained attention deficits in Han Chinese, thus supporting the hypothesis that DRD2 gene may be associated with schizophrenia phenotype [102, 103].

We found no significant links between DRD2 polymorphisms and investigated clinical parameters, except for those between rs6276, family history of schizophrenia and psychiatric disorders in general, which may be accounted for by common genetic underpinnings of psychiatric diseases and/or the fact that schizophrenia is one thereof. There exists evidence of associations between rs 1799732 and rs1800497 and the age of schizophrenia onset [31, 64], but we failed to replicate such findings, potentially attributable to population differences, random nature of these single associations or their small effect size not detected in our research due to small sample size.

Neither of the analyzed SNPs was associated with the time delay from onset to diagnosis, number of psychiatric hospitalizations, duration of longest relapse, clinical course, MMSE, PANSS, CGI-SCH or SDS scores, or symptom withdrawal after neuroleptics. However, this may result from resistance to therapy and the use of various antipsychotics, which reduces the quality of the analysis. Kang et al. [70] did not find any associations between rs1800497 and improvement of the total, positive, negative and general scores on the PANSS and the CGI-S total score after 6 weeks of amisulpride treatment. According to Ohara et al. [31], rs1799732 is not associated with the PANSS scores, while Himei et al. [41] suggest its links with the PANSS-positive score. In our study, this association did not prove significant, but close to the significance level (*p* < 0.1), which may suggest the existence of an association with a small size effect. However, further studies are necessary to verify this association. To date, there were no studies investigating the links between rs 6276 and PANSS, CGI-SCH or SDS, which precluded comparisons with our results, but the lack of direct associations with PANSS negative, CGI-SCH negative and SDS scores and DS/NDS status in our sample indicates links with the deficit syndrome only in patients with hereditary susceptibility to schizophrenia.

The main limitation of our study is a small sample size, which substantially reduces the power of this study, especially in subgroup analysis. Thus, our results should be considered as preliminary reports in need of further verification. Observed associations may be random and therefore require replication in larger samples and family studies. Sample size estimation was based on a desired power of 0.90 and *α* = 0.05 to detect medium effect size, therefore we cannot exclude a small modifier effect of the analyzed SNPs on the observed characteristics. We also analyzed a limited number of SNPs associated with DRD2. It is therefore possible that other DRD2 SNPs may have a modifying effect on the clinical course of schizophrenia. We have made every effort to ensure that the psychometric analysis is as reliable as possible, including the use of standardized scales by well-trained psychiatrists. However, symptom severity measured by the psychometric scales could be affected by various environmental factors and pharmacological therapy, and therefore the results should be interpreted carefully.

A considerable difficulty in comparing our results with previous findings is a limited number of studies investigating the relationships between the analyzed polymorphisms with schizophrenia and the fact that most of them focused on associations with the disease itself, and not necessarily its clinical course. It seems that due to its complex etiopathogenesis and varied course, the analysis of SNPs’ effect on schizophrenia phenotype should be an integral part of research on the role of genetic factors in mental disorders. More importantly, we were the first to demonstrate that rs6276 in DRD2 may be associated with the deficit syndrome in schizophrenic patients. Our results thus indicate the need for a more in-depth analysis of this polymorphism and its associations with schizophrenia. Our study also shows that research on more homogeneous patient groups, like those with family history of schizophrenia, may be beneficial in searching for disease- or symptom-related polymorphisms. This type of research could help predict the course of schizophrenia in specific patient groups.

## Conclusions

In conclusion, the results of our preliminary study do not support the hypothesis that DRD2 or ANKK1 gene polymorphisms are associated with the deficit syndrome or symptom severity in the entire population of schizophrenic patients. Analyzed polymorphisms were not associated with time delay from onset to diagnosis, number of psychiatric hospitalizations, duration of longest relapse, clinical course, MMSE, PANSS, CGI-SCH and SDS scores, or symptom withdrawal after neuroleptics. The results suggest that rs6276 in DRD2 may be associated with the deficit syndrome in patients with hereditary susceptibility to schizophrenia. Confirmation of the observed dependencies requires further research.

## Data Availability

The datasets analyzed/generated during this study are not publicly available due to patient confidentiality.

## Abbreviations

ANKK1: Ankyrin repeat and kinase domain containing 1
CGI-SCH: Clinical Global Impression-Schizophrenia scale
DRD2: Dopamine D2 receptor
DS: Patients with deficit schizophrenia
MMSE: Mini-Mental State Examination
NDS: Patients with non-deficit schizophrenia
PANSS: Positive and Negative Syndrome Scale
SDS: Schedule for the Deficit Syndrome
SNP: Single nucleotide polymorphism
